# Innovative Buccal Nanofibers for Dual Delivery of Tadalafil and Dapoxetine for Erectile Dysfunction and Premature Ejaculation Conditions

**DOI:** 10.3390/ph19040625

**Published:** 2026-04-15

**Authors:** Ali A. Alamer, Khulud A. Alsulami, Abdullah A. Alshehri, Fahad A. Almughem, Nojoud Al Fayez, Meshal K. Alnefaie, Ahmed A. Almulaifi, Alhassan H. Aodah, Essam A. Tawfik

**Affiliations:** Advanced Diagnostics and Therapeutics Institute, Health Sector, King Abdulaziz City for Science and Technology (KACST), Riyadh 11442, Saudi Arabia

**Keywords:** tadalafil, dapoxetine, buccal delivery, erectile dysfunction, electrospinning

## Abstract

**Background**: Erectile dysfunction (ED) and premature ejaculation (PE) are prevalent conditions affecting men’s sexual health, for which tadalafil and dapoxetine have shown promise in their treatment, respectively. Conventional oral dosage forms face limitations, including variable absorption and delayed onset of action. In this study, we developed electrospun nanofibers using polyvinylpyrrolidone for buccal drug delivery as an alternative dosage form to oral tablets. This route offers advantages such as easy administration, suitability for those with difficulty swallowing, particularly the elderly, and a rapid onset of action via the blood capillaries, which might improve bioavailability. **Methods:** PVP nanofibers loaded with tadalafil and dapoxetine were fabricated using a modified electrospinning procedure with the Spraybase system, where an 8% (w/v) PVP ethanol solution containing 1.5% dapoxetine and 0.5% tadalafil was electrospun under controlled conditions (800 µL/h flow rate, 15 cm distance, 0.55 mm needle, and 8–10 kV) to produce uniform fibers. **Results**: The morphology of the nanofibers was characterized using SEM, revealing smooth, uniform fibers with an average diameter of 218 ± 50 nm for drug-loaded nanofibers. This nanofibrous system also demonstrated ultra-rapid disintegration occurring within 4 ± 1 s and consistent drug loading and encapsulation efficiency for both drugs. The release profile showed a burst drug release after 15 min, which accounted for >45% for tadalafil and >50% for dapoxetine, followed by a sustained increment in the drug release that reached > 60% for tadalafil and >78% for dapoxetine after 30 min until a complete drug release (100%) for both drugs after 180 min. In vitro cytotoxicity studies on human dermal fibroblasts confirmed the safety of both medications, with cell viability exceeding 50%, at concentrations of 1.56 to 25 µg/mL for tadalafil and 4.69 to 9.38 µg/mL for dapoxetine after 24 and 48 h of incubation. **Conclusions**: These findings highlight the potential of PVP-based nanofibers as a novel buccal delivery system for the combined treatment of ED and PE.

## 1. Introduction

Erectile dysfunction (ED) affects approximately 150 million men globally, and its prevalence is anticipated to rise significantly due to an aging population, increased rates of obesity and diabetes, and expanding populations in developing countries [1]. In the United States, ED impacts over 50% of men aged 40 to 70, making it a critical public health concern. Current pharmacological treatments, particularly phosphodiesterase type 5 (PDE-5) inhibitors such as tadalafil, have demonstrated significant efficacy in the treatment of ED. Tadalafil, widely known for its role in managing ED and benign prostatic hyperplasia (BPH), exerts its effect by selectively inhibiting PDE-5, which enhances nitric oxide-mediated vasodilation in the corpus cavernosum, thus improving ED [2]. Its extended half-life of approximately 17.5 h offers sustained therapeutic effects, giving it an advantage over shorter-acting alternatives such as sildenafil [3].

Similarly, premature ejaculation (PE) affects a substantial portion of men globally, further complicating sexual health management. Dapoxetine, a selective serotonin reuptake inhibitor (SSRI) specifically designed for PE, has emerged as a novel therapeutic option. Its unique short half-life allows it to be used on demand, differentiating it from traditional SSRIs [4]. Dapoxetine increases serotonin levels, delaying ejaculation and improving sexual satisfaction [5]. Clinical studies have confirmed its efficacy, with significant improvements in intravaginal ejaculatory latency time (IELT) and overall satisfaction reported in large-scale trials [6].

The integration of tadalafil and dapoxetine into a combined therapy presents a promising approach for addressing both ED and PE, offering dual therapeutic benefits. However, despite their effectiveness, conventional oral dosage forms face challenges, including variable gastrointestinal absorption and delayed onset of action. Buccal drug delivery has emerged as a compelling alternative, providing direct access to systemic circulation while bypassing first-pass metabolism in the liver. This route enhances drug bioavailability, offers a faster onset of action, and increases patient compliance due to ease of administration [7]. However, conventional buccal dosage forms, such as films and tablets, face several challenges, including low mucosal permeability, enzymatic degradation, and rapid clearance. Thus, these limitations reduce drug retention time and absorption efficiency [8]. Therefore, highlighting the need for more advanced delivery systems to improve drug retention and enhance bioavailability.

Recent advances in electrospinning technology have enabled the fabrication of nanofibers as innovative drug delivery platforms. Electrospun nanofibers, produced by applying a high voltage to a polymeric solution, can create submicron fibers that are efficient drug carriers [9,10]. These nanofibers offer multiple benefits, including ultra-rapid drug disintegration, improved mechanical properties, and enhanced drug release, making them suitable for buccal delivery [11]. Moreover, the use of polyvinylpyrrolidone (PVP) in electrospun fibers further enhances drug encapsulation efficiency and controlled release due to its biocompatibility, biodegradability, and mucoadhesive properties, as well as its ability to dissolve freely in water and absolute ethanol [9,12,13].

In this study, we explored the design and development of tadalafil/dapoxetine-loaded nanofibers as a potential buccal delivery system for the treatment of ED and PE. The buccal route offers several advantages, including ease of administration, rapid onset of action, and the ability to bypass first-pass metabolism, which can improve the bioavailability of drugs that otherwise have low gastrointestinal absorption [7,14]. Nanofiber-based buccal delivery systems are also known for their ultra-rapid disintegration and superior mechanical properties, providing an efficient platform for the dual delivery of tadalafil and dapoxetine [11]. Although tadalafil and dapoxetine have been widely investigated in combination therapy and shown improved clinical outcomes compared to monotherapy, their co-administration has been limited to conventional oral dosage forms [15]. To the best of our knowledge, no studies have reported the co-delivery of these agents using electrospun nanofibers, particularly for buccal administration.

By utilizing electrospinning to develop a buccal delivery system, this study aims to develop and evaluate a nanofiber-based system for tadalafil and dapoxetine, with the potential to improve drug-release characteristics and enhance therapeutic performance. Incorporating both tadalafil and dapoxetine into a nanofiber matrix represents a novel approach that leverages the advantages of buccal administration while enhancing the pharmacokinetic properties of the drugs. Ultimately, this innovative delivery system has the potential to provide a more efficient, patient-friendly treatment option for ED and PE, improving the quality of life for millions of men globally.

## 2. Results

### 2.1. Morphology Assessment of Electrospun Nanofibers

**Figure 1:**
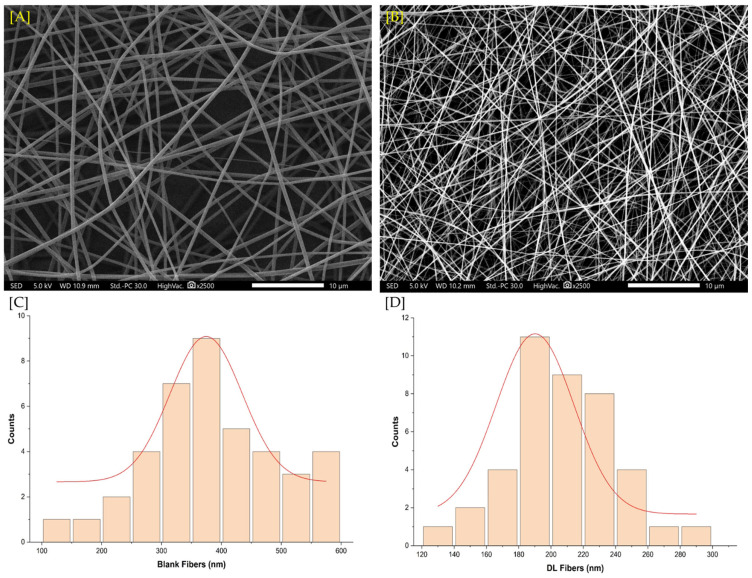
SEM images of electrospun nanofibers. (**A**) Blank nanofibers with an average diameter of 458 ± 76 nm, showing smooth surfaces without beads or pores. (**B**) Dual drug-loaded nanofibers with tadalafil and dapoxetine exhibited an increased average diameter of 218 ± 50 nm. (**C**) Diameter distribution of the blank nanofibers. (**D**) Diameter distribution of the drug-loaded nanofibers.

In this study, electrospun nanofibers were fabricated to investigate their potential as a drug delivery system for tadalafil and dapoxetine. The morphology and diameter of the nanofibers were assessed using SEM imaging to evaluate the fiber structure. SEM images (Figure 1A,B) revealed that the nanofibers had smooth surfaces, free from beads or pores, indicating uniformity and a successful electrospinning process. Diameter measurements showed that the blank fibers had an average diameter of approximately 458 ± 76 nm, whereas the dual drug-loaded fibers exhibited a narrower average diameter of 218 ± 50 nm.

### 2.2. Fourier-Transform Infrared Spectroscopy (FTIR) Analysis

**Figure 2:**
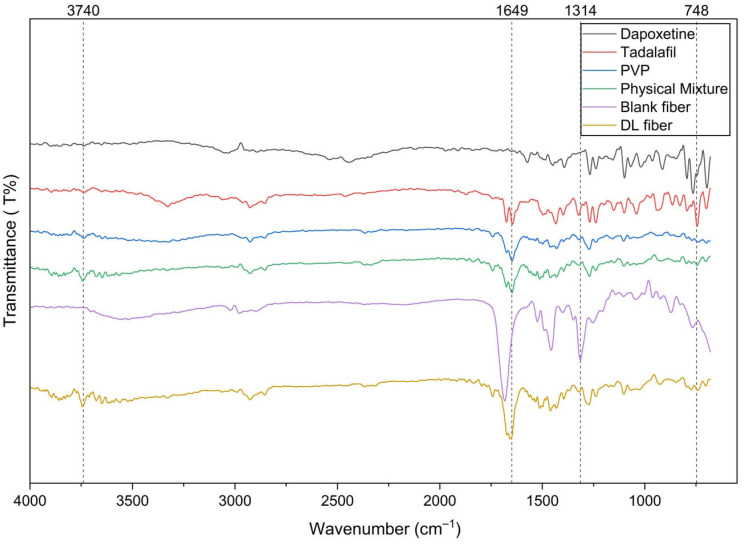
FTIR spectra of PVP, tadalafil, dapoxetine, physical mixture, the blank nanofibers (blank fiber), and drug-loaded nanofibers (DL fiber). The characteristic peaks of tadalafil and dapoxetine are visible in the spectra of the physical mixture and dual drug-loaded fibers, showing slight shifts indicative of intermolecular interactions with PVP, supporting good compatibility and stability of the drug–polymer system.

The FTIR spectra of tadalafil, dapoxetine, PVP, PM, dual drug-loaded nanofibers, and blank nanofibers were analyzed to investigate the molecular interactions and compatibility of the drugs with the polymer matrix (Figure 2). The distinctive peaks for tadalafil were observed, including amide carbonyl (C=O) stretching around 1650 cm^−1^, aromatic C-H stretching near 3050 cm^−1^, N-H bending around 1550 cm^−1^, and aromatic ring vibrations around 1600–1500 cm^−1^, which were consistent with previously reported data [16,17]. Dapoxetine exhibited characteristic peaks such as N-H stretching (amine group) between 3300–3400 cm^−1^, C-H stretching (aromatic and aliphatic) near 2900–3100 cm^−1^, C=O stretching (amide group) around 1650 cm^−1^, aromatic C=C stretching near 1500–1600 cm^−1^, and C-N stretching around 1200–1300 cm^−1^ [16,18]. In the drug-loaded fibers, there were minor shifts and band broadening in the typical PVP carbonyl area as well as in selected drug-associated bands when compared to pure components and the physical mixture. These alterations are consistent with non-covalent interactions between drugs and polymers. Tadalafil is anticipated to interact with the PVP lactam carbonyl via hydrogen bonding, whereas dapoxetine may undergo hydrogen bonding or ion-dipole interactions involving its protonated amine and the PVP carbonyl group. Because no additional absorption bands were observed, the FTIR data confirm physical integration of the drugs, with no evidence of chemical reaction.

### 2.3. X-Ray Diffraction (XRD) Analysis

**Figure 3:**
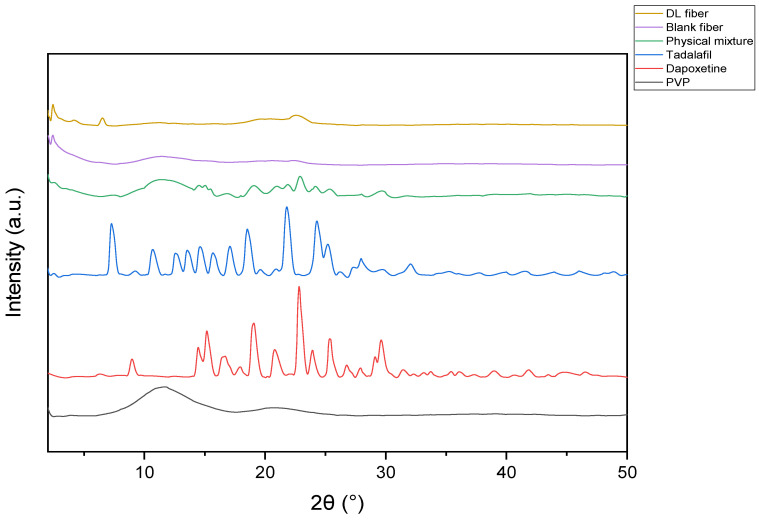
XRD patterns of PVP, tadalafil, dapoxetine physical mixture, the blank nanofibers (blank fibers), and drug-loaded nanofibers (DL fibers). The blank fiber and PVP show broad-halo patterns, indicating amorphous structures. The drug-loaded fiber shows peaks at 2θ values of 9.12° and 23.00°. Tadalafil exhibits peaks at 2θ values of 9.10°, 10.44°, 12.67°, 14.39°, and higher, indicating a crystalline phase. Dapoxetine shows peaks at 2θ: 9.12°, 14.02°, 16.72°, 19.07°, and 30.03°. The PM shows distinct peaks at 2θ, 14.05°, 19.08°, and 30.02°, confirming the presence of both drugs.

The XRD patterns in Figure 3 revealed that both the blank fibers and PVP exhibit broad halo shapes, indicative of amorphous structures. In contrast, tadalafil and dapoxetine display sharp peaks, confirming the presence of crystalline phases. Tadalafil demonstrated distinctive peaks at 2θ: 9.10°, 10.44°, 12.67°, 14.39°, 15.79°, 16.89°, 19.07°, 19.86°, 22.64°, 25.14°, 27.96° and 32.03° suggesting a highly crystalline polycrystalline phase. Dapoxetine exhibited prominent peaks at 2θ: 9.12°, 14.02°, 14.93°, 16.72°, 19.07°, 21.12°, 22.97°, 24.17°, 25.90° and 30.03°, confirming the presence of a well-crystallized phase. The PM showed sharp peaks at 2θ: 14.05°, 14.89°, 16.08°, 19.08°, 22.98°, 24.12°, 25.94°, 26.83°, and 30.02°, indicating the presence of both drugs within the polymer matrix, reflecting a well-ordered crystalline structure. The drug-loaded fibers showed intense reflections at 2θ values of 9.12° and 23.00°, corresponding to tadalafil and dapoxetine, respectively. This pattern indicates a significant decrease in long-range crystalline order and partial amorphization of the nanoscale dispersion of tadalafil and dapoxetine within the electrospun fiber matrix. Nonetheless, the current XRD data must be taken qualitatively rather than as a definitive quantification of percentage crystallinity.

### 2.4. Nanofibers Disintegration Test

**Figure 4:**
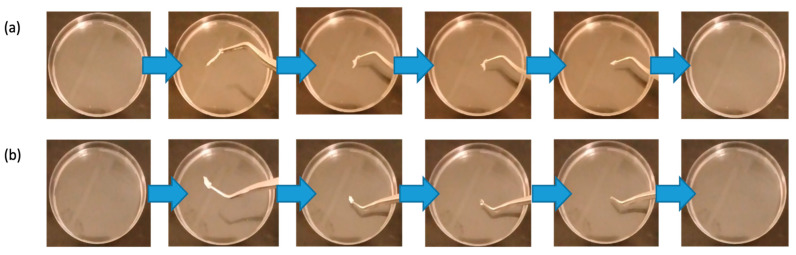
The disintegration of (**a**) blank nanofibers, 8% PVP, and (**b**) dual drug-loaded nanofibers (0.5% tadalafil, 1.5% dapoxetine) shows ultra-rapid dissolution for both systems, with times of 2 ± 1 s and 4 ± 1 s, respectively (*n* = 3).

Figure 4 demonstrates the ultra-rapid disintegration of both nanofibrous systems in PBS, highlighting their suitability for fast drug release. The blank nanofibers disintegrated in 2 ± 1 s, while the dual drug-loaded nanofibers disintegrated in 4 ± 1 s. This rapid disintegration indicated the potential effectiveness of the nanofibers for immediate drug-delivery applications and their suitability for buccal delivery.

### 2.5. High-Performance Liquid Chromatography (HPLC) Quantification

A previously described HPLC method was used to quantify tadalafil and dapoxetine. The regression equations and coefficients of determination (R^2^) were y = 11126x − 5606.4 (R^2^ = 0.9999) for tadalafil and y = 4570.7x − 2944.2 (R^2^ = 0.9999) for dapoxetine. The calibration curve demonstrated excellent linearity, confirming the method’s accuracy and successful separation of both compounds (Supplementary Materials Figure S1).

### 2.6. Drug Loading (DL), Encapsulation Efficiency (EE %), and Fiber Yield (Y) Quantification

The previously demonstrated HPLC method was used to accurately quantify the DL and EE% of the tadalafil- and dapoxetine-loaded nanofibers. The DL for tadalafil was determined to be 40 ± 1 µg/mg, with an EE% of 81 ± 2%, indicating effective drug incorporation into the nanofiber matrix. For dapoxetine, the DL was measured at 106 ± 3 µg/mg, and the EE% was 71 ± 2%, indicating successful encapsulation. The overall Y of the dual drug-loaded nanofibers was calculated at 75 ± 4%, highlighting the efficiency of the electrospinning process in producing drug-loaded fibers.

### 2.7. Drug Release Quantification

**Figure 5:**
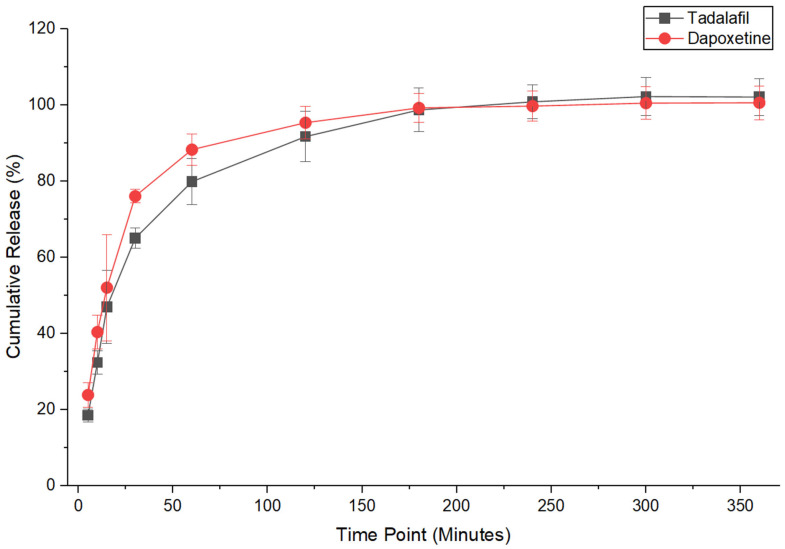
The cumulative release (%) of dual drug-loaded nanofibers over 360 min. Complete drug release was observed within 180 min for both tadalafil and dapoxetine. The results are presented as the average ± SD (*n* = 3).

The drug release profile exhibits a rapid initial release, followed by a sustained release phase and eventual plateau (Figure 5). The initial burst release occurs within the first few minutes, where more than 60% of the drug is released. This is followed by a slower, sustained release, as the remaining drug diffuses from within the nanofiber matrix. By 180 min, the release reaches a plateau, suggesting that nearly all of the drug has been fully released. This profile indicates efficient and complete drug release within 3 h, making the nanofiber system suitable for applications requiring fast and controlled drug delivery.

### 2.8. In Vitro Cell Viability Evaluation

**Figure 6:**
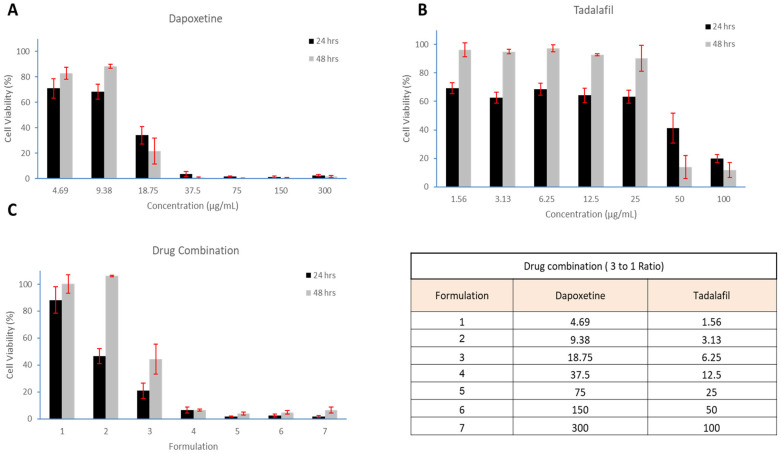
Cytotoxicity of dapoxetine (**A**), tadalafil (**B**), and their combination in a 3:1 ratio (**C**) on cell viability after 24 and 48 h of incubation. Lower concentrations of tadalafil and dapoxetine maintained high cell viability (>50%) at both time points, whereas higher concentrations of the combination showed reduced metabolic activity. Formulation details for the drug combinations are provided in panel D. Results are shown as mean ± SD (*n* = 3).

Increased concentrations of tadalafil, dapoxetine, and their combination in a 1:3 ratio were assessed for cytotoxicity in human dermal fibroblasts (HFF-1) using the MTS viability assay after 24 and 48 h incubations. The data in Figure 6 revealed that lower concentrations of tadalafil (1.56 µg/mL) and dapoxetine (4.69 µg/mL) were associated with high cell viability at both time points, with viability exceeding 60% after 24 h of incubation. Tadalafil maintained high viability across a concentration range of 25 to 1.56 µg/mL, while dapoxetine exhibited higher viability only at lower concentrations (9.38 to 4.69 µg/mL).

After 48 h, cellular viability increased to above 50% for tadalafil (25 to 1.56 µg/mL) and dapoxetine (9.38 to 4.69 µg/mL). When tested in combination at a 1:3 ratio, tadalafil and dapoxetine showed high cellular viability (˃50%) at lower concentrations (1.56:4.69 µg/mL and 3.13:9.38 µg/mL) after 24 and 48 h of incubation. However, at higher concentrations of the combination, cell viability dropped substantially, indicating increased cytotoxicity at doses above these thresholds. Overall, the combination exhibited synergistic effects, with higher cell viability at lower concentrations and lower viability at higher concentrations.

## 3. Discussion

PVP has garnered significant interest for pharmaceutical applications due to its biocompatibility, biodegradability, mucoadhesiveness, and drug-entrapment efficiency, as well as its Food and Drug Administration (FDA) approval for human use. In this study, we explored its potential use for delivering two pharmacologically distinct agents, tadalafil and dapoxetine, often used together to treat ED and PE. Tadalafil, a PDE5 inhibitor, promotes erectile function by enhancing blood flow to the penis during sexual stimulation, primarily through smooth muscle relaxation in the corpus cavernosum [19]. Due to its longer half-life, tadalafil allows for both on-demand and daily dosing, offering flexibility in treatment regimens. Dapoxetine, an SSRI, is specifically approved for PE management, delaying ejaculation by increasing serotonin levels in the synaptic cleft [6].

The combination therapy of tadalafil and dapoxetine has been shown to improve both erectile function and ejaculatory control, providing a comprehensive therapeutic approach for men with concurrent ED and PE, and enhancing sexual satisfaction and quality of life [20]. The combination of tadalafil and dapoxetine is available in some global markets only as oral tablets. However, in the absence of alternative pharmaceutical forms, using and swallowing such oral tablets can pose a significant challenge for elderly patients, as some of them suffer from dysphagia, i.e., difficulties of swallowing, leading to patients’ incompliance and dissatisfaction [21]. Therefore, developing pharmaceutical dosage forms, such as a buccal dosage form, specifically designed for the elderly, is important, as this can facilitate easier swallowing or dissolution in the mouth. The use of ultra-rapid nanofibers offers a promising alternative solution to this issue.

In this study, PVP was used to form nanofibrous drug delivery systems via electrospinning. This method provides exceptional control over drug release kinetics, complementing the nanofiber-based system developed herein, as demonstrated by Xu et al. [22]. Compared to other water-soluble polymers like Hydroxypropyl Methylcellulose (HPMC), PVP offers unique advantages, particularly its rapid dissolution and mucoadhesive properties, making it highly suitable for buccal drug delivery systems that require immediate release. Additionally, its solubility in water and absolute ethanol makes it a favorable polymer for encapsulating water-insoluble drugs. While HPMC is widely used in controlled-release formulations [23], the rapid dissolution of PVP observed in this study may enhance the bioavailability of drugs such as tadalafil and dapoxetine, potentially leading to faster therapeutic effects, particularly in the treatment of ED.

The results of this study provide significant insights into the development and characterization of electrospun nanofibers for drug delivery applications, particularly for tadalafil and dapoxetine. The morphological assessment via SEM confirmed the successful fabrication of smooth, uniform nanofibers without defects such as beads or pores, highlighting the efficacy of the electrospinning process. This was consistent with previous studies that used PVP to encapsulate a single or dual drug [24,25]. The observed decrease in the fibers’ diameter for the drug-loaded nanofibers (218 ± 50 nm) compared to the blank fibers (458 ± 76 nm) might be due to the slight increase in the voltage to control the spinning jet, which in turn has reduced the diameter of the produced fibers, as shown in the previous studies [24].

The stability of drug-loaded systems is a key consideration in drug delivery formulations, as it prevents drug crystallization or degradation during storage and ensures consistent therapeutic performance [16,17,18]. However, this FTIR result should be regarded as corroborative rather than conclusive proof of molecular compatibility. Generally, in PVP-based formulations, minor band shifts, widening, and attenuation are commonly employed to deduce hydrogen bonding or analogous noncovalent interactions between the polymer carbonyl and drug functional groups. Nonetheless, FTIR alone does not measure interaction strength and cannot independently ascertain long-term physical stability.

The XRD patterns provided further evidence of the crystalline nature of tadalafil and dapoxetine within the nanofibers. The appearance of sharp peaks for both drugs, along with broad-halo patterns for the blank fiber and PVP, confirms the presence of crystalline phases within the amorphous polymer matrix. These crystalline forms are essential for maintaining the drugs’ physicochemical stability and influencing their release kinetics. In particular, the XRD result for the dual-drug-loaded nanofibers demonstrated a low-intensity peak of tadalafil and dapoxetine, which might indicate their retention in the crystalline structures post-encapsulation, suggesting that the electrospinning process did not induce significant polymorphic transformations or degradation, which is critical for preserving their bioactivity [26,27,28]. However, the observed rapid release is likely attributable to several factors, including diminished crystallinity, PVP’s hydrophilicity, and the nanofibers’ extensive surface area.

The disintegration test revealed the ultra-rapid disintegration of the nanofibers in PBS, with complete dissolution of the drug-loaded fibers in 4 ± 1 s. This ultra-fast disintegration is a promising feature to accelerate the release kinetics of both drugs, making the nanofibers suitable for buccal drug delivery applications, where immediate disintegration and drug release are essential for rapid therapeutic efficacy via fast onset of action [29]. The rapid dissolution of PVP nanofibers is well-documented in the literature, and these findings align with previous studies highlighting the utility of PVP in fast-dissolving drug delivery systems [11,30].

The HPLC quantification of tadalafil and dapoxetine demonstrated high accuracy and precision, with excellent linearity in the calibration curves. The DL and EE% values obtained for both drugs were consistent with those reported for other PVP-based nanofiber formulations [25,31], indicating the efficiency of the electrospinning process in encapsulating both drugs. The slightly lower EE% values for dapoxetine could be attributed to differences in drug solubility and interactions with the PVP matrix during electrospinning, which may result in partial drug loss during the process. The DLs of tadalafil and dapoxetine were 40 ± 1 µg/mg and 106 ± 3 µg/mg, respectively, consistent with a 1:3 ratio between the two drugs. These results confirm that PVP is an effective polymer for encapsulating hydrophilic and hydrophobic drugs alike [11,30]. The overall Y (75%) can be considered slightly low due to fiber loss during spinning (inside the chamber) and during removal of the fibrous mat from the aluminum foil covering the metallic collector.

The drug release profile of the nanofibers was characterized by a rapid initial burst release, followed by a more controlled, sustained release. The release of more than 60% of the drug within the first few minutes suggests that a significant portion of the drug is located near the surface of the nanofibers, allowing for immediate dissolution upon contact with the release medium. The subsequent slower release phase is attributed to the gradual diffusion of the remaining drug from within the fiber matrix. This biphasic release pattern is ideal for providing both an immediate therapeutic effect and sustained drug availability, which can be advantageous in managing conditions that require fast onset and prolonged action [32]. This finding is advantageous, especially during buccal application of the drug-loaded fibers, where PVP has mucoadhesive properties and may remain in the buccal cavity for longer than a minute. The used release medium (20% PBS, pH 6.8, and 80% ethanol) is considered unusual. This was due to the low solubility of the dual-drug-loaded nanofibers in 100% PBS (as indicated by visual turbidity), so we reduced the PBS concentration and increased the ethanol concentration until a clear solution was obtained. Additionally, this release medium helped maintain sink conditions for this release study.

Finally, the in vitro cell viability tests using HFF-1 cells confirmed the safety of the nanofiber system at lower concentrations of tadalafil and dapoxetine. High cell viability (>50%) was observed at tadalafil concentrations of 1.56 to 25 µg/mL and dapoxetine concentrations of 4.69 to 9.38 µg/mL after 24 and 48 h of incubation. However, at higher concentrations, cell viability decreased, particularly for dapoxetine, suggesting dose-dependent cytotoxicity. When the drugs were combined in a 3:1 ratio, cell viability remained high at lower concentrations, further supporting the formulation’s safety for potential clinical use. These findings align with previous studies that demonstrated the low cytotoxicity of PVP-based nanofibers and the importance of optimizing drug concentrations to ensure both efficacy and safety [25].

Overall, the cell viability results are considered important, as they highlight the need to tailor the final formulation to safer drug concentrations. The combination demonstrated high cell viability at lower concentrations, but a substantial decrease in viability at higher concentrations indicates potential cytotoxicity. These insights are critical for guiding formulation strategies, emphasizing the importance of maintaining drug concentrations within the safer, lower range and potentially adjusting the ratio to optimize both efficacy and cellular tolerance. While the current 3:1 ratio of dapoxetine to tadalafil was selected based on commercially available oral tablets, these findings suggest that this ratio may need to be re-evaluated to maintain a higher safety profile for the attended final product, i.e., buccal delivery system. This is because drugs rapidly absorb into the bloodstream through capillaries in the buccal cavity, which theoretically would require less dosing than oral tablets. Hence, a further in vivo pharmacokinetics/pharmacodynamics study would be required to support the development of a safe and effective final formulation.

## 4. Materials and Methods

Polyvinylpyrrolidone (PVP) polymer with a molecular weight of approximately 1,300,000, HPLC-grade dimethylformamide (DMF), ethanol (≥99.5%), acetonitrile, phosphate-buffered saline (PBS) tablets, and chloroform were purchased from Sigma-Aldrich (St. Louis, MO, USA). The PBS solution was prepared by dissolving 5 PBS tablets in 1 L of distilled water, and the pH was adjusted to 6.8 with 5 M HCl. Tadalafil EP (Lot No. D5071-17-005M1M), referred to as tadalafil throughout the manuscript, was gifted a sample from the stability lab in Riyadh, Saudi Arabia. Dapoxetine hydrochloride (CAS No. 129938-20-1), referred to as dapoxetine, was purchased from Tokyo Chemical Industry Pvt. Ltd. (Telangana, India), and triethanolamine was obtained from Loba Chemie (Mumbai, India). Distilled water was produced using a Milli-Q Millipore system (Billerica, MA, USA).

### 4.1. Preparation of PVP Nanofibers Loaded with Tadalafil and Dapoxetine

In this study, the Spraybase^®^ electrospinning system (Dublin, Ireland) was used to fabricate PVP nanofibers loaded with tadalafil and dapoxetine using a modified electrospinning procedure [33]. PVP polymer solution was prepared by dissolving 8% (*w*/*v*) PVP powder in ethanol and stirring for 2 h until fully dissolved. Dapoxetine (1.5% *w*/*v*) was added and stirred for an additional hour, followed by the addition of 0.5% (*w*/*v*) tadalafil to obtain an 8:1.5:0.5 polymer–drug ratio. The mixture was further stirred for one hour to ensure uniform mixing. A control solution containing 8% (*w*/*v*) PVP without drugs was used as a blank. Electrospinning was performed at room temperature and 30–45% relative humidity. The syringe pump was set to a flow rate of 800 µL/h, with a 15 cm distance between the needle and the collector, using a 0.55 mm inner-diameter needle. Voltages of 10 kV and 8 kV were applied to form stable jets for the drug-loaded and blank fibers, respectively, and the fibers were collected onto an aluminum-foil-covered metallic collector.

### 4.2. Scanning Electron Microscopy (SEM) Imaging

The morphology and surface structure of drug-free and dual-drug-loaded nanofibers were examined using a JSM-IT500HR SEM (JEOL Inc., Peabody, MA, USA), and fiber diameters were measured using ImageJ software (Version 1.46r, National Institutes of Health, Bethesda, MD, USA). Approximately 20 fibers were analyzed for each system, and an accelerated voltage of 5 kV was applied. Nanofibers were collected on aluminum foil and coated with a 2 nm platinum layer using a JEC3000FC auto fine coater.

### 4.3. Fourier-Transform Infrared Spectroscopy (FTIR) Analysis

Fourier-Transform Infrared Spectroscopy (FTIR) analysis was performed using a Thermo Smart ATR IS20 Spectrometer (Thermo Fisher Scientific, Waltham, MA, USA) with a spectral resolution of 4 cm^−1^ and 32 scans per sample. Samples were analyzed within the wavenumber range of 4000 to 600 cm^−1^, including the tadalafil–dapoxetine-loaded PVP nanofibers, a physical mixture (PM) containing the same raw materials in powder form, and the blank nanofibers. Approximately 5 mg of each sample was used for analysis, and the FTIR spectra were plotted using OriginPro^®^ 2021 software (OriginLab Corporation, Northampton, MA, USA).

### 4.4. X-Ray Diffraction (XRD) Analysis

X-ray diffraction (XRD) analysis was carried out using a Rigaku Miniflex 300/600 instrument (Tokyo, Japan) with Cu Kα radiation (1.5148 Å), an operational voltage of 40 kV, and an applied current of 15 mA. Samples including tadalafil, dapoxetine, PVP, the PM, and the blank and dual-drug-loaded nanofibers were scanned in the 2θ range of 2° to 50° at a scanning speed of 5°/min.

### 4.5. Nanofibers Disintegration Test

The disintegration of the dual drug-loaded and blank nanofibers was evaluated using a modified method (Alshaya et al., 2022) [33]. Square portions of nanofiber mats (3 mg) were placed in pre-warmed 5 mL PBS (pH 6.8, 37 °C) within Petri dishes. Disintegration was observed at different time intervals using a shaking incubator (Excella E24 Incubator Shaker Series, New Brunswick Scientific Co., Enfield, CT, USA). Results were reported as the average ± standard deviation (SD) of three independent replicates.

### 4.6. High-Performance Liquid Chromatography (HPLC) Quantification

The concentrations of tadalafil and dapoxetine were determined using a Waters e2695 HPLC system with a Waters^®^ 717 plus autosampler, Waters 600 binary pump, and Waters 2489 UV detector (Waters Technologies Corporation, Milford, MA, USA). Separation was achieved using isocratic elution with a mobile phase composed of 1% triethanolamine (adjusted to pH 3.9 with formic acid) and methanol (30:70). Chromatographic analysis was performed on an XBridge C_18_ column (5 µm, 4.6 × 250 mm) at 20 °C with a 1 mL/min flow rate. The injection volume was 10 µL, and detection was at 260 nm. Stock solutions of tadalafil and dapoxetine were prepared in 80% ethanol and 20% PBS (pH 6.8), and calibration curves were constructed using serial dilutions ranging from 500 to 0.92 µg/mL. Retention times (Rts) were 5 min for tadalafil and 6 min for dapoxetine.

### 4.7. Drug Loading (DL), Encapsulation Efficiency (EE %), and Fiber Yield (Y) Quantification

To quantify drug loading (DL) and encapsulation efficiency (EE%) of the tadalafil–dapoxetine nanofibers, at least three samples (5 ± 0.1 mg) were dissolved in 10 mL of a solvent mixture containing 20% (*v*/*v*) PBS (pH 6.8) and 80% (*v*/*v*) ethanol. The solutions were left at room temperature for 6 h under constant stirring to ensure complete dissolution of the nanofibers. The drug concentrations were then quantified using the developed HPLC method. The following equations were used to calculate EE% and DL:

$$EE\%\;=\;\frac{Actual\;entrapped\;drug\;amount}{Theoretical\;entrapped\;drug\;amount}\;\times \;100$$

$$DL=\frac{Entrapped\;drug\;amount}{Actual\;nanofibers\;amount}$$

The fiber yield (Y%) was determined using the following equation:

$$Y\%\;=\;\frac{Actual\;nanofibers\;amount}{Theoretical\;nanofibers\;amount}\;\times \;100$$

The theoretical nanofiber amount was calculated based on the solid materials in the spinning solution.

### 4.8. Drug Release Quantification

The release profiles of tadalafil and dapoxetine from the nanofibers were assessed using 20 ± 0.1 mg samples in 15 mL of a pre-warmed solvent mixture (20% PBS, pH 6.8, and 80% ethanol) in glass vials. This release medium was used to ensure complete dissolution of both drugs and to maintain sink conditions, rather than using a saliva-mimicking physiological medium. It was initially observed that 100% PBS exhibited noticeable turbidity and incomplete dissolution, largely due to the limited aqueous solubility of tadalafil [34]. Therefore, the hydroalcoholic medium was employed to guarantee complete drug solubility and facilitate quantitative comparison of release from the nanofibers [33]. The solvent mixture was then incubated at 37 °C in a shaking incubator (100 RPM; Excella E24 Incubator Shaker Series, New Brunswick Scientific Co., Enfield, CT, USA). At designated time intervals (5 to 180 min), 1 mL samples were taken and replaced with fresh solvent. Drug concentrations were measured using HPLC. The cumulative drug release (%) was calculated using the following equation:

$$Cumulative\;Release\;\%\;=\;\frac{Cumulative\;drug\;amount}{Theoretical\;drug\;amount}\;\times \;100$$

### 4.9. In Vitro Cell Viability Evaluation

The in vitro cell viability of tadalafil and dapoxetine was evaluated using a colorimetric MTS assay (CellTiter 96^®^ Aqueous One Solution, Promega, Southampton, UK) following 24 and 48 h exposure to human fibroblast cells (HFF-1; ATCC-SCRC-1401), which were obtained from the American Type Culture Collection (ATCC; Manassas, VA, USA). This assay was designed to determine the optimal concentration of tadalafil and dapoxetine, and their combination that would exert a therapeutic effect while maintaining cell safety. The method was adapted from Alamer et al. [25].

HFF-1 cells were sub-cultured in Dulbecco’s Modified Eagle Medium (DMEM) containing 10% FBS (*v*/*v*), 100 μg/mL streptomycin, and 100 U/mL penicillin. Once cells reached 80–90% confluency, they were detached using trypsin and counted with the trypan blue exclusion test. Cells were seeded into 96-well plates at a density of 1.5 × 10^4^ cells/well and incubated overnight at 37 °C in 5% CO_2_. Serial dilutions of tadalafil (100–2 µg/mL), dapoxetine (300–5 µg/mL), and their combination (1:3 ratio) were added to the wells and incubated for 24 or 48 h. The negative control contained cells in DMEM only, while the positive control was incubated with 0.2% Triton X-100.

After incubation, the medium was aspirated, the wells were gently washed with PBS, and 100 µL of fresh DMEM was added. MTS reagent (20 µL) was then added, and plates were incubated for an additional 4 h. The absorbance of the formazan product was measured at 490 nm using a microplate reader (Cytation 3, BIOTEK Instruments Inc., Winooski, VT, USA). Cell viability was calculated using the following equation:

$$CellViability\;\%=\;\frac{\left(S-T\right)}{\left(H-T\right)}\;\times \;100$$

where *S* represents the absorbance of treated cells, *T* is the absorbance of the positive control, and *H* is the absorbance of the negative control. Results are reported as the average ± SD of independent triplicate measurements.

### 4.10. Statistical Analysis

The EE%, DL, Y%, and drug-release quantification experiments were conducted in at least 3 replicates, with results expressed as the mean ± SD. Results are presented as the average ± standard deviation (SD) from independent triplicates. release profile, XRD patterns, and FTIR spectra were performed using OriginPro^®^ 2021 software (OriginLab Corporation, Northampton, MA, USA). Data analysis of the EE%, DL, Y%, disintegration test, and in vitro cell viability was performed using Microsoft Excel 2019, and graphs were plotted for the in vitro cell viability experiment.

## 5. Conclusions

The development of tadalafil- and dapoxetine-loaded PVP nanofibers provides a promising alternative to conventional oral dosage forms for the treatment of ED and PE. The electrospinning process successfully produced smooth, defect-free nanofibers with enhanced structural properties. FTIR and XRD analyses confirmed the compatibility of the drugs within the nanofiber matrix, while rapid disintegration in PBS indicated the potential for rapid drug release via buccal administration. HPLC analysis confirmed effective DL and EE (>70% for both drugs), with full drug release after 3 h. In vitro cytotoxicity testing demonstrated the formulation’s safety at lower drug concentrations, supporting its potential clinical application. Overall, these findings suggest that PVP-based nanofibers could provide an efficient, patient-friendly drug-delivery platform for men with both ED and PE, thereby improving therapeutic outcomes and patient quality of life.

## Data Availability

The original contributions presented in the study are included in the article; further inquiries can be directed to the corresponding authors.

## Supplementary Materials

The following supporting information can be downloaded at: https://www.mdpi.com/article/10.3390/ph19040625/s1. Table S1. Calibration Curve Data for Tadalafil and Dapoxetine. Figure S1. Calibration curves of Tadalafil (a) and Dapoxetine (b): concentration (µg/mL) vs AUC. Table S2. Calculated limit of detection (LOD) and limit of quantification (LOQ) for Tadalafil and Dapoxetine. Table S3. Precision study results for Tadalafil and Dapoxetine. Table S4. Accuracy study results showing recovery (%) for Tadalafil (a) and Dapoxetine (b). Figure S2. HPLC chromatogram of Tadalafil (RT = 5 min) and Dapoxetine (RT = 6 min). Figure S3. HPLC chromatogram of blank sample.
