# Supplementary material for: Innovative Buccal Nanofibers for Dual Delivery of Tadalafil and Dapoxetine for Erectile Dysfunction and Premature Ejaculation Conditions
Source: Pharmaceuticals (Basel). 2026 Apr 15;19(4):625. doi: 10.3390/ph19040625 (PMC13119127; doi:10.3390/ph19040625)
Supplement: Supplementary file 1 [file pharmaceuticals-19-00625-s001.zip › pharmaceuticals-4221700-supplementary.pdf]

Supplementary Material

HPLC Method Validation Data

1. Calibration Curve and Linearity

The calibration curves were constructed over the concentration range of 3.125–200 µg/mL for both tadalafil and dapoxetine.

Table S1. Calibration Curve Data for Tadalafil and Dapoxetine. The area under the curve results for tadalafil and dapoxetine.

Concentration (µg/mL)	Tadalafil AUC	Dapoxetine AUC
**200**	2217286	910961
**100**	1114601	455765
**50**	547005	224160
**25**	269152	109294
**12.5**	131449	53689
**6.25**	65444	26590
**3.125**	31612	12924

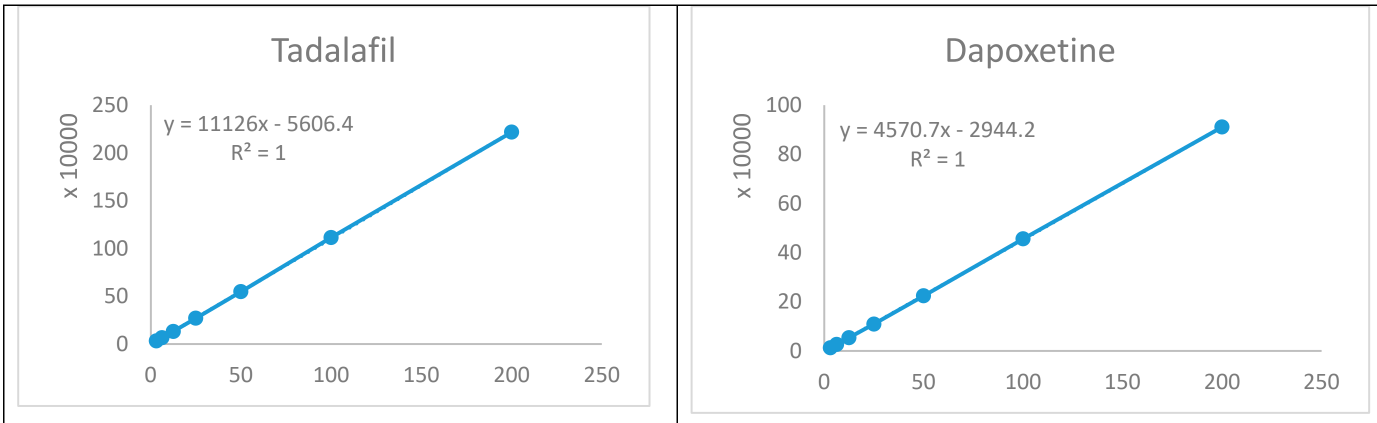

(a)	(b)
Regression equation for Tadalafil:<br>$y = 11126x - 5606.4$<br>$R^2 = 0.9999$	Regression equation for Dapoxetine:<br>$y = 4570.7x-2944.2$<br>$R^2 = 0.9999$

Figure S1. Calibration curves of Tadalafil (a) and Dapoxetine (b): concentration (µg/mL) vs AUC.

2. Limit of Detection (LOD) and Limit of Quantification (LOQ)

Table S2: Calculated limit of detection (LOD) and limit of quantification (LOQ) for Tadalafil and Dapoxetine.

Blank AUC values	Mean blank response	Standard deviation (SD)
4017, 4386, 4550	4317.67	271.6

LOD and LOQ were calculated according to ICH guidelines using:

LOD = 3.3 × SD/slope

LOQ = 10 × SD/slope

Calculated values:

Drug	Slope	LOD (µg/mL)	LOQ (µg/mL)
**Tadalafil**	11126	0.081	0.244
**Dapoxetine**	4570.7	0.196	0.594

The calculated LOD and LOQ values for tadalafil were 0.081 and 0.244 µg/mL, respectively, while for dapoxetine they were 0.196 and 0.594 µg/mL, respectively, indicating adequate sensitivity of the developed HPLC method. According to the ICH guidelines, the LOQ should be lower than the lowest concentration of the calibration range to ensure reliable quantification. In this study, all LOQ values were significantly below the lowest calibration point (3.125 µg/mL), confirming the method's acceptability and suitability.

3. **Precision (Repeatability, n = 3)**

Table S3. Precision study results for Tadalafil and Dapoxetine.

Tadalafil AUC values	Dapoxetine AUC values
948085, 929335, 939335	351264, 348468, 349242
Mean = 938918.33	Mean = 349658
Standard deviation ≈ 9397	Standard deviation ≈ 1443
%RSD = 1.00%	%RSD = 0.41%

The %RSD values were below 2%, meeting the ICH acceptance criteria and demonstrating excellent repeatability of the developed HPLC method.

4. **Accuracy (Recovery Study, n = 3)**

Table S4. Accuracy study results showing recovery (%) for Tadalafil (a) and Dapoxetine (b).

Recovery was assessed by comparing the AUCs of spiked nanofiber samples with those of the corresponding standard solutions.

<u>For Tadalafil (a)</u>

Standard AUC	Spiked AUC	Recovery (%)
**405721**	405721	100.00
**405822**	406122	100.07
**406221**	405801	99.90

Mean recovery = 99.99%

<u>For Dapoxetine (b)</u>

Standard AUC	Spiked AUC	Recovery (%)
**152553**	152553	100.00
**153557**	153110	99.71
**152653**	152632	99.99

Mean recovery = 99.90%

The recovery values (98–102%) meet the ICH acceptance criteria for assay methods, confirming the method's accuracy and the absence of matrix interference in the nanofiber formulation.

5. Specificity

Chromatographic analysis showed well-resolved, distinct peaks for tadalafil and dapoxetine, with no interfering peaks from the nanofiber matrix at their respective retention times, confirming method specificity.

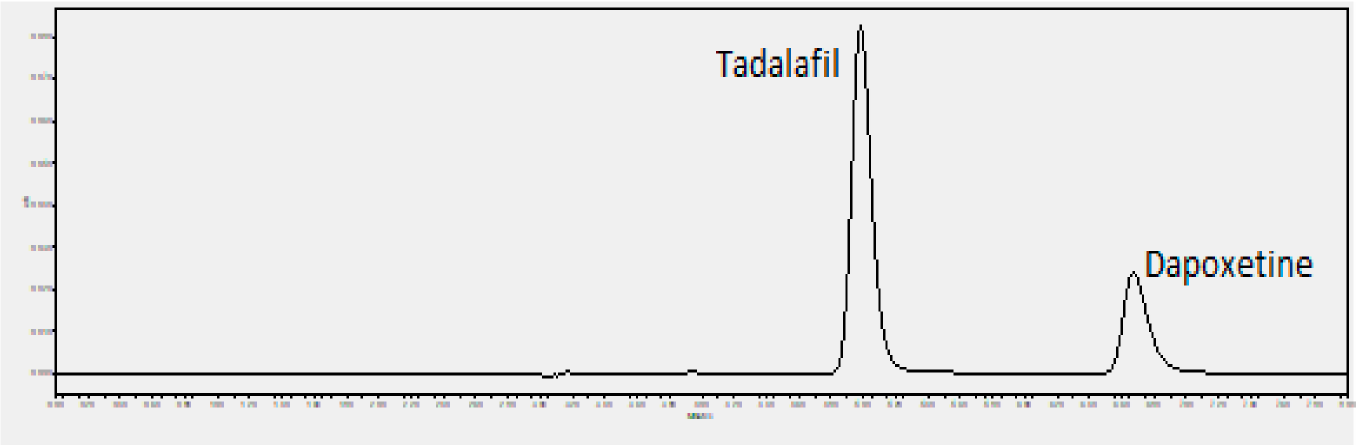

Figure S2. HPLC chromatogram of Tadalafil (RT = 5 min) and Dapoxetine (RT = 6 min).

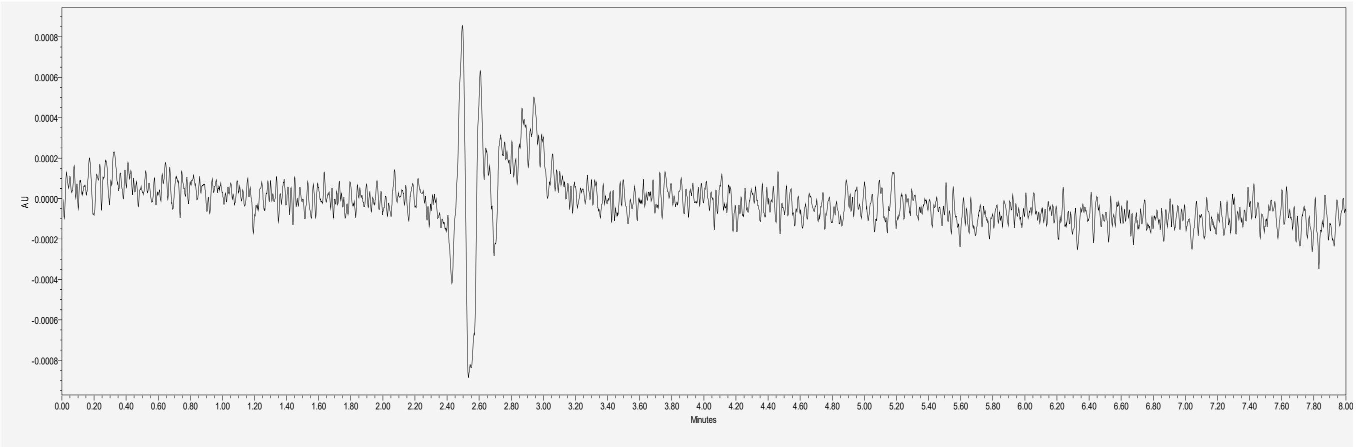

Figure S3. HPLC chromatogram of blank sample.
